# Microarray-Based Gene Expression Analysis of Hepatocellular Carcinoma

**DOI:** 10.2174/138920210791233063

**Published:** 2010-06

**Authors:** Thorsten Maass, Ioannis Sfakianakis, Frank Staib, Markus Krupp, Peter R Galle, Andreas Teufel

**Affiliations:** Department of Medicine I, Johannes Gutenberg University, Mainz, Germany

**Keywords:** HCC, systems biology, bioinformatics, oncogenomics.

## Abstract

Microarray studies have successfully shed light on various aspects of the molecular mechanisms behind the development of hepatocellular carcinoma (HCC), such as the identification of novel molecular subgroups and the genetic profiles associated with metastasis and venous invasion. These experiments, mainly comprising genome wide profiling, potentially represent the basis of novel targeted therapeutic strategies in HCC. In response, we summarize the multiple reported expression profiles in HCC associated with HCC development, novel subgroups, venous invasion and metastasis.

## INTRODUCTION

Hepatocellular carcinoma is one of the most common cancers worldwide and the third most common cause of death due to cancer [1, 2]. Several etiological factors have been recognized, including viral hepatitis B (HBV) infection, hepatitis C (HCV) infection and alcohol abuse, although the molecular pathogenesis is not well-known [3, 4]. Few patients are eligible for surgery, the only curative treatment. The severity of this disease, the lack of good diagnostic markers and treatment strategies, as well as clinical heterogeneity have rendered the disease a major challenge [3]. Thus, a better understanding of the molecular pathogenesis of these tumors, through genome-wide gene expression studies, is crucial to overcoming these difficulties.

## HEPATOCELLULAR CARCINOMA

In most cases HCC arises in cirrhotic livers secondary to various environmental risk factors [5]. However, HCC may also develop in both normal liver, and abnormal non-cirrhotic liver. HBV and HCV infections are the major risk factors, leading to cirrhosis and HCC. Other risk factors for liver cancer include alcohol abuse and aflatoxin B1 (AFB1) exposure [6].

Each of above scenarios involves different genetic and epigenetic alterations, chromosomal aberrations, gene mutations and differential activation/inhibition of molecular pathways [5]. Data from microarrays demonstrate different genetic profiles depending on the origin of the liver tumor, whether it arises in cirrhotic or non-cirrhotic liver, or within tumors in patients with HBV-infection, HCV-infection or alcohol abuse [5]. 

## APPLICATIONS OF MICROARRAY TECHNOLOGY IN HCC RESEARCH

Microarray technology has been widely applied to identify the molecular and genomic mechanisms in liver tumorigenesis. Over the past decade, a great variety of gene expression profile studies have been performed.

These studies have succeeded in further exploring the molecular basis of HCC development in a number of ways, namely by identifying specific gene expression patterns discriminating HCC from non-tumor liver. This reveals consistent gene expression patterns associated with etiological factors, histological phenotypes, and clinical phenotypes, as well as unveiling novel subtypes of HCC previously unrecognized by conventional methods [3].

## STUDIES DISCRIMINATING HCC FROM NONTUMOR LIVERS

The genomic signatures of HCC can be used to help characterize the molecular changes responsible for HCC development. With respect to clinical practice this will be particularly valuable in the development of novel tumor markers, with higher sensitivity than Alpha-Fetoprotein (AFP). Furthermore, large scale analysis of gene expression may be of significance in promoting the identification of novel therapeutic targets. For example, constantly upregulated genes in HCC and their corresponding protein may be potential targets for a therapeutic intervention with a blocking antibody. Those strategies have been successfully introduced in other cancers e.g. Her2neu blockade by Trastuzumab in breast cancer.

Lau *et al*. [7] were the first to use microarray technology to compare gene expression profiles of HCC and non-HCC liver tissues. Since then, multiple comparative studies have been published. Microarray analyses between HCC and non-tumor tissues allowed the identification of a number of potential genetic networks, deregulated in the context of liver carcinogenesis. Among these, Wnt-signaling pathway, p53-signaling pathway, TGF-β, MAPK, IGF-2 and the Jak/Stat pathway were demonstrated to be differentially regulated by means of microarray experiments [8-12]. Looking at individual genes, many of them have been identified as demonstrating significantly altered expression between HCCs and non-tumor livers. However, confirming each and every one of these regulated genes from one microarray based analysis to the next remains difficult. Therefore, it seemed more useful to focus on superimposed categories of genes. Some of the most differentially expressed categories of genes between HCC and non-tumor livers are genes related to cell cycle progression, RNA splicing, protein degradation, cell adhesion, metabolic enzymes, detoxification, immune response, extracellular matrix and cytoskeleton, DNA damage repair system, and apoptosis, and also cytokines, growth factors, oncogenes, tumorsupressors, and GTP-binding proteins [8, 9, 13-16].

Many genes were significantly upregulated in HCC tissue. Examples include those required for cell cycle progression whose expression levels correlated with cellular proliferation rates, genes expressed in endothelial cells [13] regulating the composition of the extracellular matrix and cytoskeleton [8, 14], and genes involved in DNA repair systems [8, 15]. Imprinted genes, and genes encoding secreted proteins [16], were also significantly upregulated. Furthermore, genes associated with immune response [8, 13, 14, 16], coding for detoxification proteins [9, 10, 14], encoding metabolic enzymes [8, 9, 13, 14, 16], and many plasma protein coding genes [8, 9, 13, 14] were significantly down-regulated in HCC tissues compared to adjacent non-tumorous liver. 

Besides the general mechanisms and genetic signatures associated with HCC development, it was noticed that the underlying chronic liver disease may significantly impact on the molecular mechanisms leading to HCC development. From a clinical point of view, this may account for the considerable variance in risk of developing HCC among patients with different underlying chronic liver diseases [17, 18]. Currently most data analyzing the genetic signatures of HCC from patients with diverse underlying liver diseases focused on patients with HBV versus HCV infection [19-23]. The first genome-wide microarray study comparing HBV- with HCV-related HCC was performed by Okabe *et al*. [9]. Interestingly, genes involved in drug metabolism and carcinogen detoxification were reported to be differentially regulated between HCV-based and HBV-based HCC. An increased expression of phase I modification enzymes (CYP2E, AKP1C4, EPHX1, and FMO3) were observed exclusively in HCV-positive HCCs, whereas expression of phase II-conjugated reaction enzymes (UGT1A1, UGT2G10, and GPX2) were preferentially repressed in HBV-positive HCCs. Phase I and phase II reactions are metabolic pathways responsible for drug metabolism in the liver but also the metabolization of xenobiotic carcinogens [24]. Phase I enzymes convert several pro-carcinogens to activated metabolites. Thus, the overexpression of Phase I modification genes suggests that this enhanced expression leads to a greater contribution of carcinogenic metabolites to the mechanisms of HCV-specific hepatocarcinogenesis. In contrast, decreased expression of enzymes catalyzing phase II-conjugated reactions suggests that the decreased expression of detoxification enzymes may be involved in the mechanisms of HBV-specific tumorigenesis. This hypothesis was furthermore supported by Iizuka *et al*. [10] who also reported an increased expression of phase I enzymes (CYP2E) in HCV-induced HCCs compared to HBV-induced HCCs and a significant reduction of detoxification-related genes in HBV-associated HCC. Moreover, the same study reported, that a number of immune response–related genes (IFI27, OAS1, ISG15, and IFI14) were up-regulated in HCV-associated HCCs compared to HBV-associated HCCs and to non-tumorous liver. Genes that may promote metastasis (MMP-9, VEGF, HMMR, TACSTD1, and MCAM) were up-regulated in HBV-associated HCC. Finally, this study was the first to report that imprinted genes such as H19 and IGF-2 were increased in HBV-associated HCC in comparison to both HCV-associated HCC and non-tumorous liver. Delpuech *et al*. [8] tried to summarize the comparison of HBV- versus HCV- associated HCC by describing the involvement of different cellular pathways. In HCV-specific cancer a more heterogenous gene expression pattern was identified with an overexpression of the TGF-β induced genes. 

In conclusion, these results suggested that HBV and HCV lead to the development of liver cancer by different mechanisms. Key molecular mechanisms suggested by currently available publications include a blockade of the detoxification system [10], alteration of immune response mechanisms, inflammation, and specific genetic pathways such as TGF-β pathway. The deregulation of imprinted genes furthermore suggested imprinting has a role to play in the development of HCC as well. 

## GENE EXPRESSION PROFILES ASSOCIATED WITH DIFFERENT HISTOLOGICAL GRADES OF HCC AND PRECANCEROUS DYSPLASTIC NODULES

It has repeatedly been proposed that tumorigenesis is a multistep process that often involves a complex series of multiple and diverse genetic alterations. Best analyzed are the multi-step changes during the development of colorectal carcinoma where at least seven sequential genetic alterations are proposed [25]. 

Concerning HCC progression multiple studies have analyzed global transcription levels in the context of different histological grades and precancerous nodules (Table **1**). Wurmbach *et al*. [11] reported that the onset of HCC (dysplasia vs early HCC) was associated with major changes in gene expression as summarized in Table **1**. Pathway analysis showed that early HCC may be distinguished from dysplasia by a significant down-regulation of components of the Toll-like receptor pathway, Jak/STAT pathway, TGF pathway, and the insulin-signaling pathway. In contrast, components of the Wnt-signaling pathway were up-regulated. Progression of cancer was characterized by strong up-regulation of proliferation related genes. Many studies used Edmonson-Steiner histopathological classification system [26] to discriminate well- to poorly differentiated HCCs (Edmonson grade GI to IV). Yu *et al*. [27], reported that the transition from liver cirrhosis to GI or GII HCC is associated with up-regulation of molecular chaperone genes including CCT5, HSPCG, CCT3, HSPCA, DNAJBII, HSPBI, and TOMM. Generally molecular chaperones, primarily members of the Hsp70 and Hsp90 families, regulate many key components of survival and apoptotic pathways [28]. Hsp70 has been reported to be a sensitive marker for the differential diagnosis of early HCC from a precancerous lesion or a non-cancerous liver [29]. This work further suggested that a deterioration of immune surveillance and apoptosis occurs at an early stage of HCC development, as many apoptosis and major histocompatibility complex related genes were down-regulated during the transition from liver cirrhosis to GI or GII HCC [27]. Nam *et al*. [30] reported a series of 120 genes to be classifiers for distinguishing between low- (LGDN) and high grade dysplastic nodules (HGDN) and also between HGDN and GI HCC. Accuracy of these classifiers were 95%. Moreover, a 120-gene molecular signature in HCC specimens could discriminate effectively (with 91% classification accuracy) between GI-GII and GII-GIII HCC as well. Finally, Okabe *et al*. [9] compared GI to GII or GIII HCC and identified decreased levels of apoptosis related proteins (Table **1**) in moderately-to-poorly differentiated HCC, supporting the hypothesis that a reduced rate of apoptosis may be a major characteristic of tumor progression.

## DISCOVERING NOVEL SUBTYPES OF HCC

Several studies have demonstrated that HCC may be subdivided into at least two prognostically diverse subgroups based on gene expression patterns. Characterizing these molecular subgroups by microarray studies is essential since these subgroups may not be distinguishable by conventional histology but still potentially respond differently to treatment. Thus, a clearer and better characterization of these diverse HCC subgroups may be crucial to entering an era of personalized HCC treatment, with the promises of higher efficacy, with the added benefit of fewer side effects. Lee *et al*. [31] identified a novel subtype of HCC that shared gene expression patterns with fetal hepatoblasts. Furthermore, expression of well-known markers of hepatic oval cells, and the early progenitors of adult liver stem cells enabled the authors to suggest that this subtype of HCC may arise from hepatic progenitor cells. Interestingly, based on the hepatoblast specific gene expression signature, HCC individuals could be subdivided into 2 subpopulations with significant differences in overall survival. HCC individuals who expressed the hepatoblast signature had a poorer prognosis. The complete signature however comprised 907 genes. A recent study by Yamashita *et al*. [32] classified HCCs in 4 groups based on EpCAM expression and patients’ serum levels of α-fetoprotein (AFP). The same group had previously described EpCAM to be an early biomarker of HCC. 35% of HCC cases express EpCAM [33]. Gene expression analysis in these subgroups showed that each of these 4 subgroups had a unique expression pattern with features resembling various stages of hepatic lineages such as hepatic stem cell-like HCCs, bile-duct epithelium-like HCCs, hepatocyte–like HCCs, and mature hepatocyte-like HCCs. These 4 subgroups were each associated with a distinct prognosis. Hepatic stem cell-like HCCs and hepatocyte-like HCCs had poor prognosis, while hepatocyte-like HCCs and bile-duct epithelium- like HCCs had fair and good prognosis, respectively [32]. Together these recent developments in microarray analysis of larger HCC datasets provide new insights into the potential cellular origin of HCCs. They demonstrate the presence of prognostically relevant subgroups, which were distinguishable only by means of genetic signatures, not by histology alone. However, these studies describe completely different and at the same time large signatures. Thus, the challenge remains to isolate key identifiers of the diverse subgroups that are present in multiple datasets, and also to reduce the number of genes relevant to a gene signature, as this will promote bringing these signatures into clinical practice.

## GENE EXPRESSION PROFILE STUDIES ASSOCIATED WITH METASTASIS

Metastasis of HCC fundamentally changes the prognosis of the tumor as surgery is no longer an option, and treatment of metastasized HCC is limited to palliation. Thus, identifying genetic signatures associated with metastasis and subsequently being able to target the corresponding genetic processes would mean a significant improvement in HCC therapy. 

Diverse DNA microarray studies have reported many genes implicated in tumor metastasis [34-39]. RhoC-GTPase, Granulin-epithelin precursor (GEP), Vimentin (VIM), and Platelet-derived growth factor receptor alpha (PDGFRA) were identified as being over-expressed in highly metastatic HCC and therefore may be considered for future strategies targeting the metastatic potential of HCC [34-39]. Besides, strong GEP expression was associated with large HCCs, venous infiltration, and early intrahepatic recurrence. A recent microarray study reported 4 genes (HLA-DRA, HLA-DRB1, HLA-DG, and HLA-DQA) of the same family of HLA class II antigens to be down-regulated in HCCs with early intrahepatic recurrence [40]. In particular, low levels of HLA-DR protein in tumor cells correlated positively with transcription levels of the HLA-DRA gene. This tended to be associated with early intrahepatic metastasis and advanced tumor stage [40].

## GENE EXPRESSION PROFILE STUDIES ASSOCIATED WITH INTRAVENOUS INVASION

Venous invasion (VI), particularly portal venous invasion (PVI) is a hallmark of the intrahepatic spread of HCC cells, and therefore of poor outcome [41]. A better understanding of the underlying genetic mechanisms may in the future at least help to limit the growth and metastasis of HCC, and help keep patients in disease stages eligible for surgery or transplantation for longer periods of time.

Genome-wide gene expression profile studies identified a number of genes associated with venous invasion (Table **2**). Ho *et al*. [42] compared gene expression profiles between HCC specimens with VI and HCC specimens without VI, and identified 14 genes essentially related to venous invasion. Among these genes, 3 were related to cell growth (SLC4A7, RAB38, and RYR), while individual genes were involved in biosynthesis processes in human liver and muscle (AMPD3), and in oxidative-DNA damage (8-OHdG). Interestingly, the genetic signatures of these 14 genes in HCC specimens could effectively discriminate patients at high risk of VI development or recurrence after curative hepatectomy [42]. Chen *et al*. [13] reported 91 genes that significantly correlated with the presence of VI. Most of these genes had functions associated with cell proliferation, while one gene was involved in the breakdown of extracellular matrix (MMP-14), and another one had antiangiogenic and antiproliferative activity (ADAMTS1). Okabe *et al*. [9] identified 151 genes associated with VI. This group of genes, with mostly unknown functions (110 genes), contained 3 members of small GTPase-related genes (RhoC, RhoGAP8 and ARHGEFG). RhoC-GTPase was found to be up-regulated in HCC specimens with VI compared to HCC specimens without VI, while ARHGAP8 and ARHGEFG were preferentially down-regulated in VI tissues in comparison to non-VI tissues. Over-expression of RhoC in HCC specimens was recently reported to be strongly correlated with HCC venous invasion and metastasis [36]. Although the function of ARHGAP8 and ARHGEFG is unknown, ARHGAP8 is thought to inhibit the Rho-signaling pathway; hence reduced expression of ARHGAP8 may also results in Rhomediated tumor invasion. These findings led the authors to assume that controlling the Rho-pathway may suppress tumor invasion and subsequent metastasis [9]. Tsunedomi *et al*. [43] identified 35 differentially-regulated genes related to portal venous invasion (PVI). These genes were involved in apoptosis and stress response (SGK, API5, GADD45B), cell proliferation (RAN, NUDC), oncogenesis (DDXI), and signal transduction (RHO6). Interestingly, a gene called inhibitor of DNA binding 2 (ID2), was found to be significantly down-regulated in HCC specimens with PVI. This is in accordance with a recent report [44] demonstrating that ID2 protein levels decrease in parallel with HCC progression. ID proteins are negative regulators of cell growth and cell differentiation [45-48].

## PREDICTION STUDIES TO DEVELOP PREDICTORS FOR HCC OUTCOME

Gene signatures in HCC tissues or noncancerous hepatic surrounding tissues, highly associated with prediction of clinical outcome (such as recurrence or overall survival after surgical resection of tumor) have been designated “predictors”. Prediction studies aim to build a predictor and to evaluate its performance on an independent set of HCC patients [49]. Therefore, these studies use a training-validation approach in which a predictor is built on the basis of information from training samples and its predictive power is evaluated on an independent sample set [49]. Several kinds of prediction studies have been performed and are summarized below.

## STUDIES PREDICTING RECURRENCE AFTER SURGICAL RESECTION OF THE TUMOR

The poor outcome of HCC patients results partly from post-surgical recurrence of the primary tumor. Prediction studies identified multiple gene signatures in cancerous or noncancerous hepatic tissues which may serve as predictive tools to determine post-surgical recurrence. These signatures may aid in identifying patients most likely to benefit from surgery. Several studies used venous invasion as a cancer characteristic to build a predictor for recurrence after surgical resection of the tumor. Vascular invasion (VI) in pathology specimens is a well-known unfavorable prognostic factor for HCC recurrence [42]. Based on the hypothesis that a gene expression profile for VI in HCC specimens can predict post-surgical recurrence, Ho *et al*. [42], identified a gene signature in cancerous hepatic tissues which consists of 14 genes essentially related to vascular invasion (VI). This signature can provide an accurate discrimination between patients with high and low risk of VI development and recurrence after curative hepatectomy. Budhu *et al*. [50], reported a 17-gene signature expressed in noncancerous hepatic tissues with venous metastasis, capable of significantly discriminating patients who are likely to experience recurrence within 3 years after surgical hepatectomy (79% sensitivity). This predictive signature, largely contributed to by inflammatory and immune response-related genes, can also predict venous metastases developed several years after resection of the tumor. A recent study [51] revealed overexpression of Aurora kinase B, a chromosome passenger protein kinase, in HCC tissues, to be the most significant predictor of the aggressive recurrence of HCC after surgical hepatectomy. Japanese authors used early intrahepatic recurrence (IHR) as a clinical outcome to build a predictor [49]. Iizuka *et al*. [52] reported a gene signature of 12 genes related to immune response, expressed in cancerous tissues, which can effectively detect HCC patients at high risk of early IHR within 1 year after curative surgery. Accuracy in validation test was 92%. Kurokawa *et al*. [53] identified a 20-gene signature expressed in HCC tissues, capable of predicting early IHR within 2 years after HCC resection. This gene signature, mainly consisting of E-cadherin and immune response-related genes, predicted early IHR with 73% accuracy. Somura *et al*. [54] reported a 3-gene signature expressed in HCC areas to be predictor of early IHR within 1 year after surgery. This 3-gene- combination (HLA-DRA, DDX17, and LAPTM5) predicted early IHR with 81% accuracy in the validation group. Finally, Claudin-10 (CLDN10), a gene encoding integral membrane protein and tight junction strands, was found to be over-expressed in recurrent HCC tumors after curative hepatectomy [37]. Therefore, the authors proposed that CLDN10 could serve as a molecular marker for metastatic potential, and recurrence, after curative resection of the tumor.

## STUDIES PREDICTING OVERALL SURVIVAL AFTER SURGICAL HEPATECTOMY

Few expression studies have focused on the potential role of gene signatures as predictors of overall survival after surgical resection of the tumor. Lee *et al*. [55] reported that a 406-gene expression signature can effectively divide HCC patients into 2 subpopulations, with significant differences in survival. Genes associated with cell proliferation, apoptosis, histone modification, and ubiquitination provided the most accurate discrimination between subpopulations. A novel survival-related subpopulation of HCC patients which was associated with a poor prognosis, and was characterized by a gene expression profile resembling that of fetal hepatoblast, was added to the former classification [31]. 

In another study, a signature of 111-Met regulated genes enabled prediction of survival in HCC patients [56]. The presence of the Met-gene signature defined a subpopulation of HCC patients who had a worse survival rate compared to patients in whom Met-signature was absent.

## STUDIES PREDICTING CHEMOTHERAPEUTIC RESPONSE IN PATIENTS WITH ADVANCED HCC

Chemotherapeutic treatment is often associated with severe side effects and in light of limited prognosis, treatment with the most effective drug may be crucial. Thus, microarray based prediction of effectiveness of drugs in individual patients may be the key to a more specific treatment. In the pre-sorafenib era, combination chemotherapy with 5-fluorouracil (5-FU) and interferon-α (IFN-α) has been demonstrated to be beneficial in some patients [51, 55]. Kurokawa *et al*. [53] revealed that a 63-gene-signature could effectively (85% accuracy in validation group) select patients eligible for this treatment. These 63 predictive genes included some genes that had previously [57-64] been reported to be associated with sensitivity to 5-FU (MMP-9, HK2, TNFSF6) or IFN-α (CALM-1, ENPEP, BIRC4, OASL). Moriyama *et al*. [65] reported that gene expression of 27 predictive genes could select patients for combination 5-FU/ IFN-α therapy. Among these genes, 10 were associated with sensitivity to 5-FU alone. However, with sorafenib being the first drug showing a significant improvement in survival and other targeted therapy concepts being investigated in clinical trial, the data relating to 5FU is likely to become less relevant. Rather, the hepatologic community needs data on treatment response to sorafenib, depending on the genetic background of the tumor. This is particularly important since the Asian trial [66] demonstrated a significantly lower benefit for treatment with sorafenib than previously described [66]. Data demonstrating this has yet to be generated. 

## POTENTIAL BIOMARKERS FOR EARLY HCC DIAGNOSIS

HCC is a challenging malignancy which possesses a dismal outcome, partly due to the poor identification of early–stage HCC. The combination of serum AFP and ultrasonography is widely used as a screening procedure for the presence of HCC in high risk populations [67, 68]; however it is not reliable for the early detection of HCC [69, 70]. Clearly, there is an urgent need for novel serologic HCC biomarkers that have a sufficient sensitivity and specificity to aid in the early diagnosis of HCC. DNA microarray studies attempted to identify novel serological markers, by targeting genes that are characteristically highly expressed in HCC tissues and whose products are secreted in serum. Jia *et al*. [69] identified 5 genes (GPC3, PEG10, MDK, SERPINI1, and QP-C), encoding known serum proteins, over-expressed in most HCC cases, including those with normal AFP. A combined score using these 5 genes can accurately differentiate non-cancerous hepatic tissues (with 100% accuracy) and HCCs (with 71% accuracy). Lee *et al*. [71] reported that cystatin B (CSTB) could serve as a potential serum marker in HCC. CSTB is specifically over expressed in most HCCs and is also elevated in the serum of a large proportion of HCC patients. The specificity and sensitivity of CSTB were 85,5% and 53,1% respectively, in distinguishing between patients with HCC and those with non-malignant chronic liver disease. It will be of significant interest having future studies which aim to combine these diverse markers, in order to maximize their diagnostic accuracy. 

## CONCLUSION AND PERSPECTIVES

Microarray studies have successfully shed light on various aspects of the molecular mechanisms of hepatocellular carcinoma such as tumor development, the identification of novel molecular subgroups, and also genetic profiles associated with metastasis and venous invasion. One of the major problems in large microarray analysis experiments remains the comparability between different data sets. The challenge remains to extract a few essential genes from these large lists of regulated genes that will aid in early detection, subclassification and determining prognosis of HCC. Successful targeting may ultimately lead to further advances in preventive medicine and targeted personalized therapy of HCC. Further support in this direction may come from advances in other high-throughput technologies in genomics, especially next generation sequencing, proteomics, and also in the development of novel bioinformatics algorithms. Even more, integrating the diverse data from microarray data, genome wide mutation screens, and the increasingly available proteome data will provide further support. Combining this with clinical data will present novel perspectives on the diagnosis and treatment of HCC in the next decade. 

## Figures and Tables

**Table 1:** Genes Expression Profile Studies Associated with Different Histological Grades of HCC and Precancerous Dysplastic Nodules

Study	Upregulated	Downregulated
Wurmbach *et al*. [11], Transition from dysplasia to early cancer	Genes involved in cell cycle, protein biosynthesis and RNA processing, cell division, DNA replication, protein modification, ubiquitin cycle, chromatin remodeling, wnt pathway	Genes involved in cytokine-cytokine receptor interaction, Ca signaling, Jack/STAT pathway, Toll-like receptor pathway, TGF-β pathway, insulin growth pathway
Wurmbach *et al*. [11], Transition from early to advanced cancer	Genes involved in cell proliferation, DNA repair and replication (PRIM1, PRIM2), ubiquitin cycle, RNA processing and cell cycle regulation (ASPM, PTTG1, CCNB1, CDKN2C, CDKN2A)	
Yu *et al*. [27], Transition from cirrhosis to well-to-moderately differentiated HCC	Molecular chaperone genes (CCT5, HSPCG, CCT3, HSPCA, DNAJB11, HSPB, TOMM) and tumor-related genes (EFNA1, MDK, RBM17, FDPS, p8, LASS2 anti-metastasis gene)	Tumor suppressor and apoptosis-related genes (STAT1, IGFBP7, THY28 and DDB1), genes related to the major histocompatibility complex (HLA-DRB3, HLA-DPA1, HLA-DRA), liver specific genes
Yu *et al*. [27], Transition from well-to-moderately differentiated to poorly differentiated HCC	Tumor progression-related genes (LAPT5, CD74, UBEIL, PRG1, DKK1, LAMB1) and metastasis or invasion genes (including S100A11, S100A6, RGS19/P1, TIMP2)	
Yu *et al*. [9], Transition from well-to-moderately differentiated to poorly differentiated HCC	Apoptosis related proteins (TNFSF10, TNFSF14, GADD34, CFLAR, CLU, CASPC, phosphatidylserine receptor)	

**Table 2:** Genes Related to Venous Invasion of HCC

Study	Upregulated	Downregulated
Ho *et al*. [42]	Tumor specific antigen (MAGE 9), oxidative DNA damage (8-OHdG), proliferation regulator (TAFB4), growth factors (G-CSFR-1), cell growth (SLC4A7, RAB38, RYR), and potent mitogen inhibitor (Thrombin inhibitor)	KIAA1441
Chen *et al*. [13]	Genes associated with proliferation and in breakdown of extracellular matrix (MMP-14)	Genes encoding liver-specific proteins (CYPs) and antiangiogenic activity (ADAMTS1)
Okabe *et al*. [9]	Gene encoding small GTPase enzyme (RhoC-GTPase)	Genes encoding small GTPase enzyme (ARHGAP8, ARHGEFG)
Tsunedomi *et al*. [43]	Genes involved in apoptosis and stress response (API5), cell proliferation (RAN, NUDC), and oncogenesis (DDX1)	Genes involved in apoptosis and stress response (SGK, GADD45B) and signal transduction (RHO6)
